# A Novel M7G-Related MicroRNAs Risk Signature Predicts the Prognosis and Tumor Microenvironment of Kidney Renal Clear Cell Carcinoma

**DOI:** 10.3389/fgene.2022.922358

**Published:** 2022-06-24

**Authors:** Peng Hong, Huifang Du, Ming Tong, Qingfei Cao, Ding Hu, Jiaji Ma, Yanyang Jin, Zizhi Li, Weichao Huang, Guangquan Tong

**Affiliations:** ^1^ Department of Urology, The First Hospital of Jinzhou Medical University, Jinzhou Medical University, Jinzhou, China; ^2^ Affiliated First Hospital, Nanchang University, Nanchang, China

**Keywords:** kidney renal clear cell carcinoma, prognostic signature, m7G, microRNA, tumor immune microenvironment, The Cancer Genome Atlas

## Abstract

**Background:** M7G modification is extremely vital for the development of many cancers, especially tumor immunity. M7G modification is a novel functional regulator of miRNA, and the researches on m7G-related miRNAs in kidney renal clear cell carcinoma (KIRC) are still insufficient. This research aims to establish a risk signature on the foundation of m7G-associated miRNAs, which can precisely forecast the prognosis of KIRC patients.

**Methods:** Transcriptome data and clinical data used in this study come from The Cancer Genome Atlas database. Our team utilized univariable Cox, Lasso and multivariable Cox analyses to construct a m7G-associated miRNAs risk signature that can forecast the prognosis of KIRC patients. Kaplan-Meier method, time-dependent receiver operating characteristic (ROC) curve, and the independent analysis of risk signatures were employed to verify the predictability and accuracy of the risk signature. Subsequently, based on CIBERSORT, ESTIMATE and ssGSEA algorithms, we speculated the potential impact of the proposed risk signature on tumor immune microenvironment. Ultimately, by virtue of the risk signature and tumor immunity, the hub genes affecting the prognosis of KIRC patients were screened out.

**Results:** Our team established and verified a prognostic signature comprising 7 m7G-associated miRNAs (*miR-342-3p, miR-221-3p, miR-222-3p, miR-1277-3p, miR-6718-5p, miR-1251-5p, and miR-486-5p*). The results of the Kaplan-Meier survival analysis revealed that the prognosis of KIRC sufferers in the high-risk group was often unsatisfactory. The accuracy of the prediction ability of the risk signature was verified by calculating the area under the ROC curve. Univariate-multivariate Cox analyses further showed that this risk signature could be utilized as an independent prognosis-related biomarker for KIRC sufferers. The results of the immune analysis revealed that remarkable diversities existed in immune status and tumor microenvironment between high-risk and low-risk groups. On the foundation of the proposed risk signature and other clinical factors, a nomogram was established to quantitatively forecast the survival of KIRC sufferers at 1, 3 and 5 years.

**Conclusion:** Based on m7G-related miRNAs, a risk signature was successfully constructed, which could precisely forecast the prognosis of sufferers and guide personalized immunotherapy for KIRC patients.

## Introduction

Renal cell cancer takes up 2%–3% of adult malignancies, second only to prostate carcinoma and bladder carcinoma in urinary system cancers, whereas it is the most deadly malignancy of the urinary system (Humphrey et al., 2016; Rouprêt et al., 2021). Kidney renal clear cell carcinoma (KIRC) is the most commonly seen histologic type of renal cell cancer, taking up approximately 80% (Pandey and Syed, 2022). According to Cancer Statistics, there will be about 79,000 new cases of kidney carcinoma in the United States in 2022, with approximately 13,920 new deaths due to such disease (Siegel et al., 2022). In recent years, the incidence of KIRC has been increasing, and the age of KIRC patients has become younger (Aragon-Ching et al., 2019). Partial nephrectomy and radical nephrectomy are still the most important and effective treatment methods, whereas about 20%–30% KIRC patients encounter recurrence after surgical treatment, which reduces the 5-year survival rate to approximately 23% (Maclennan et al., 2012; Campbell et al., 2017; Hsieh et al., 2017; Speed et al., 2017). Although some new therapeutic methods have been developed, such as targeted drug therapy and immunotherapy, the clinical outcomes of advanced KIRC are still poor (Richter and Dvořák, 2018). The poor prognoses of patients with KIRC are still a clinical challenge, with delayed diagnoses and high metastasis rates being important factors inducing the poor prognoses of KIRC sufferers. Consequently, it is pivotal to propose a novel biomarker and molecular target for KIRC patients.

RNA methylation is a posttranscription modification extensive in eukaryotic cells and prokaryotes, and methylation denotes the transfer of a methyl group from one active methyl compound to another (Zhang et al., 2021). Depending on methylation sites such as m6A, m5C, m7G, and 2-O, RNA methylation can be divided into various methylation modifications (Hori, 2014; Boccaletto et al., 2018; Zhang et al., 2021). N7-methylguanosine (m7G) is a modification of the 7th N of RNA guanine with a methyl group (Alexandrov et al., 2002). As post-transcriptional modifications, m7G plays a momentous function in the initiation of miRNA biogenesis, cell migration, stability, translation, immunogenicity and many other biological processes such as Writers (methyltransferases), Erasers (demethylases), Readers (binding proteins) (Tomikawa, 2018; Zhang et al., 2021). M7G modification exists not only on mRNAs, but also on miRNAs (Pandolfini et al., 2019). M7G methylation complexes include *METTL1* (methyltransferase like 1) and *WDR4* (WD Repeat Domain 4) (Lin et al., 2018). M7G modification affects tumor progression in many ways, especially in tumor immunity. For example, *METTL1* promotes the processing of let-7e microRNA through m7G methylation, and then regulates the let-7e miRNA/*HMGA2* axis, thus inhibiting the occurrence and development of colon cancer (Baradaran Ghavami et al., 2017; Pandolfini et al., 2019; Liu et al., 2020). *METTL1* enhances the translation of oncogenic mRNA through m7G tRNA modification to promote the development of intrahepatic cholangiocarcinoma (Dai et al., 2021). Abnormal translation mediated by *METTL1*/*WDR4*-mediated m7G tRNA modification promotes the progression of head and neck squamous cell carcinoma (Chen J. et al., 2022).

MicroRNAs (miRNAs) are short single-stranded ncRNA molecules (19–25 nucleotides), which regulate gene expression after transcription and are indispensable in many biological processes like differentiation, apoptosis, drug resistance, proliferation, and metastasis (Bartel, 2009). Some recent scientific studies have unveiled that abnormally expressed miRNAs can affect the progression of many diseases to a certain extent, including stomach cancer, bladder cancer, lung adenocarcinoma, prostate cancer and colorectal cancer, etc. (Inamoto et al., 2018; Sharma and Baruah, 2019). MiRNAs are highly stable in a variety of biological samples, including tissue, serum and saliva, and their detection is easy as well (Jung et al., 2010). These characteristics provide favorable conditions for miRNAs to become candidate biomarkers for the diagnosis of cancer or precancerous lesions and prognosis. M7G modification is a novel functional regulator of miRNA, and the researches on m7G-associated miRNAs in KIRC are still insufficient. We need to further clarify the potential association between m7G-associated miRNAs and KIRC, investigating the significance of m7G-associated miRNAs as prognostic biomarkers in KIRC patients and providing ideas for developing new treatment methods for KIRC.

In this research, our team utilized The Cancer Genome Atlas (TCGA) public database to construct and verify a risk signature on the foundation of m7G-associated miRNAs, which could accurately forecast the prognoses of KIRC sufferers. It is proven that this risk signature can be utilized as an independent prognosis biomarker for KIRC patients, and it can also facilitate individualized immunotherapy for KIRC patients clinically. Finally, we used our risk signature and other clinical factors to establish a nomogram that could quantitatively forecast the overall survival (OS) rate of KIRC sufferers.

## Materials and Methods

### Transcriptome Data and Clinical Data Acquisition of KIRC Patients

**FIGURE 1:**
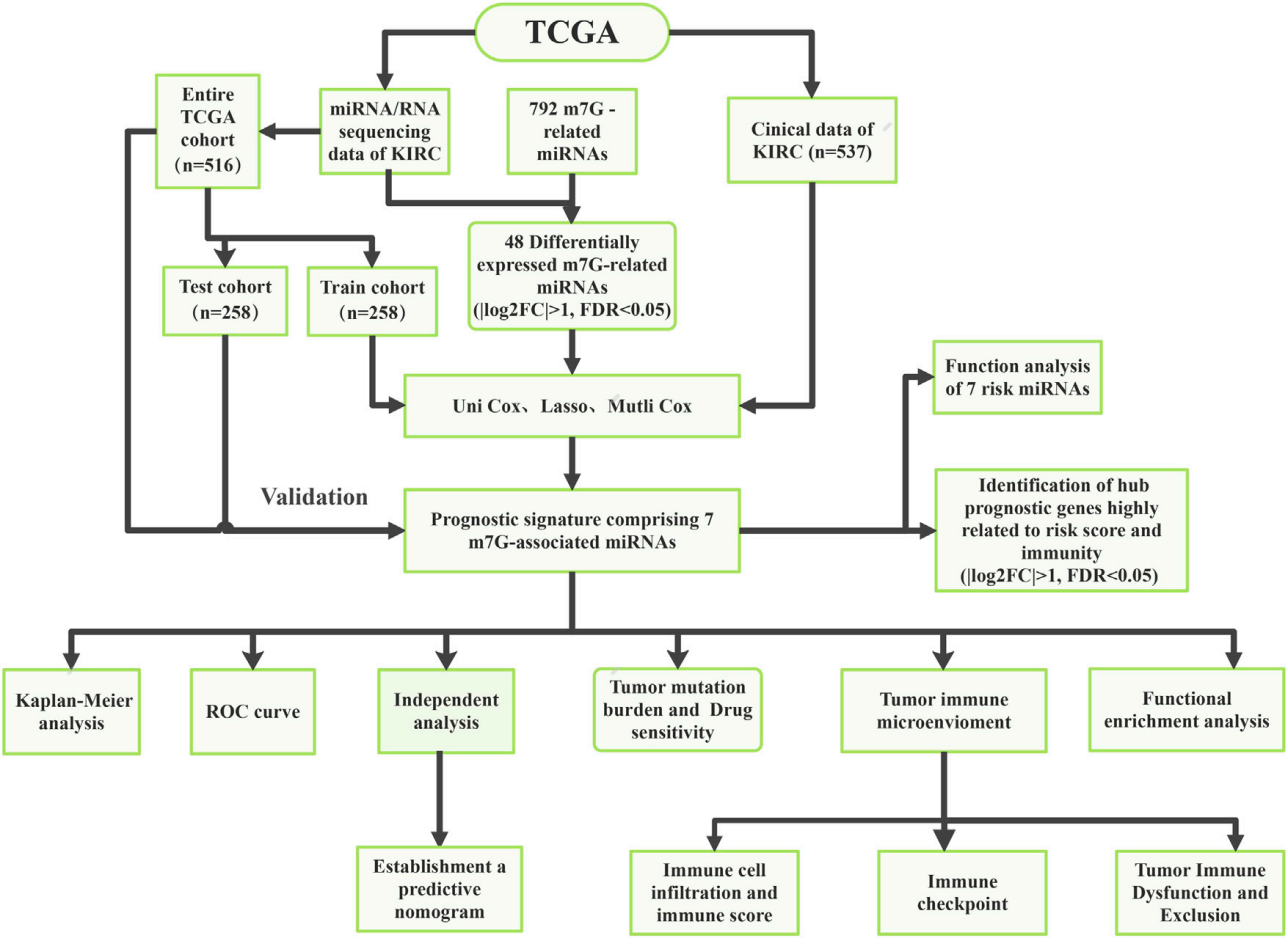
The flow chart of this study.

The flowchart of the present research is displayed by Figure 1. The clinical data and sequencing data of KIRC patients in the present research were obtained from the TCGA database (Creighton et al., 2013). Mature miRNA sequences were obtained in Fasta format from miRNAbase and used for miRNA annotation. The miRNA sequencing data (Isoform Expression) of 545 KIRC tissular specimens and 71 healthy renal tissular specimens were obtained. The RNA sequencing data (TCGA-KIRC, HTSeq-Counts) of 539 KIRC tissular specimens and 72 healthy renal tissular specimens were also obtained from the TCGA database. In addition, the corresponding clinical data were acquired from the TCGA database. TCGA pan-cancer data covering 33 tumors were downloaded from the UCSC Xean browser, and 18 cancer types along with their corresponding normal tissue samples were utilized for further study.

By excluding some samples with incomplete data, a total of 516 KIRC samples with complete miRNA/RNA sequencing data and clinical data were used for subsequent analysis (Table 1). The 516 KIRC samples were randomized 1:1 into two cohorts: the training cohort (*n* = 258, for the construction of the m7G-related miRNAs risk signature) and the testing cohort (*n* = 258, for the verification of the risk signature).

**TABLE 1:** Clinicopathological characteristics of 516 ccRCC patients in TCGA-KIRC cohort.

Characteristic	Type	Total TCGA cohort (N = 516)	Testing cohort (*N* = 258)	Training cohort (*N* = 258)	*p* value
Age	≤65	342 (66.28%)	174 (67.7%)	168 (64.86%)	0.5558
	>65	174 (33.72%)	83 (32.3%)	91 (35.14%)	
Gender	FEMALE	181 (35.08%)	90 (35.02%)	91 (35.14%)	1
	MALE	335 (64.92%)	167 (64.98%)	168 (64.86%)	
Grade	G1	13 (2.52%)	6 (2.33%)	7 (2.7%)	0.836
	G2	218 (42.25%)	103 (40.08%)	115 (44.4%)	
	G3	202 (39.15%)	104 (40.47%)	98 (37.84%)	
	G4	75 (14.53%)	40 (15.56%)	35 (13.51%)	
	GX	5 (0.97%)	3 (1.17%)	2 (0.77%)	
	Unknow	3 (0.58%)	1 (0.39%)	2 (0.77%)	
Stage	Stage I	253 (49.03%)	117 (45.53%)	136 (52.51%)	0.1585
	Stage II	55 (10.66%)	30 (11.67%)	25 (9.65%)	
	Stage III	123 (23.84%)	59 (22.96%)	64 (24.71%)	
	Stage IV	82 (15.89%)	49 (19.07%)	33 (12.74%)	
	unknow	3 (0.58%)	2 (0.78%)	1 (0.39%)	
T	T1	21 (4.07%)	10 (3.89%)	11 (4.25%)	0.921
	T1a	132 (25.58%)	59 (22.96%)	73 (28.19%)	
	T1b	106 (20.54%)	53 (20.62%)	53 (20.46%)	
	T2	54 (10.47%)	27 (10.51%)	27 (10.42%)	
	T2a	9 (1.74%)	4 (1.56%)	5 (1.93%)	
	T2b	4 (0.78%)	3 (1.17%)	1 (0.39%)	
	T3	5 (0.97%)	3 (1.17%)	2 (0.77%)	
	T3a	119 (23.06%)	66 (25.68%)	53 (20.46%)	
	T3b	53 (10.27%)	25 (9.73%)	28 (10.81%)	
	T3c	2 (0.39%)	1 (0.39%)	1 (0.39%)	
	T4	11 (2.13%)	6 (2.33%)	5 (1.93%)	
M	M0	406 (78.68%)	195 (75.88%)	211 (81.47%)	0.1328
	M1	78 (15.12%)	47 (18.29%)	31 (11.97%)	
	MX	30 (5.81%)	14 (5.45%)	16 (6.18%)	
	unknow	2 (0.39%)	1 (0.39%)	1 (0.39%)	
N	N0	228 (44.19%)	115 (44.75%)	113 (43.63%)	0.1884
	N1	17 (3.29%)	12 (4.67%)	5 (1.93%)	
	NX	271 (52.52%)	130 (50.58%)	141 (54.44%)	

### Screening of Differentially Expressed m7G-Related MicroRNAs

From previous literature reports, we obtained two m7G-related genes (*METTL1* and *WDR4*) that were confirmed to be associated with m7G modification on miRNA (Lin et al., 2018; Dai et al., 2021; Zhang et al., 2021). We used the TargetScan (https://www.targetscan.org) online database to predict the upstream miRNA of m7G-related genes and obtained m7G-related miRNAs. The “edgeR” package of R program was used to select differentially expressed m7G-associated miRNAs in KIRC tissue samples and normal kidney tissue samples (selection criteria: |log2 Fold Change (FC)| > 1, False Discovery Rate (FDR) < 0.05) (Liu et al., 2021). The expression of differentially expressed m7G-related miRNAs was shown by the heatmap.

### Establishment and Validation of the Predictive Risk Signature Based on m7G-Related MicroRNAs

Univariable Cox analyses were utilized to assess the prognosis significance of the m7G-related miRNA with differential expression (*p* < 0.05). Lasso regressive analyses were further used to optimize the selection of prognostic m7G-related miRNAs (Tibshirani, 1997), and then these most representative prognostic m7G-related miRNAs were analyzed by multivariate Cox regression. After the above analysis, specific risk miRNAs and their corresponding coefficients were given, and a prognosis signature on the foundation of m7G-associated miRNAs was constructed. The risk scoring for every KIRC sufferer can be computed as:

$$\text{Risk score}=\sum_{\text{i}=1}^{\text{n}}coef\left(miRNA\right)\;*\;Exp\left(miRNA\right)$$

Taking the median risk score of the training cohort as the threshold, KIRC patients in each cohort (training cohort, test cohort, and full TCGA cohort) were divided into the risk_high_ group and risk_low_ group for subsequent analysis and validation. The Chi-square test was employed to verify the association between clinic features and risk scoring. The Kaplan-Meier (K-M) survival curve (Log-rank test) was employed to evaluate OS diversities between these 2 groups. The accurateness of the modeling method was evaluated by calculating the area under the ROC curve (AUC) values. Univariable-multivariable Cox proportional risk analysis was utilized to evaluate whether risk scores and clinical features were independent prognostic biomarkers for KIRC patients.

### Establishment and Calibration of Nomogram

On the foundation of risk scoring and other clinical independent prognostic factors, a nomogram risk identification model was established *via* R-package “rms”. Nomogram can quantify the factors that affect the prognosis of patients with KIRC and predict the prognosis of patients with KIRC quantitatively. Subsequently, calibration curves were developed to illustrate the predictive power of the established nomogram. The calibration curve describes the calibration of each model according to the consistency between the predicted survival time of KIRC patients and the observed survival time of KIRC patients. The *y*-axis represents the actual survival time of KIRC patients. The *x*-axis represents the predicted survival time of KIRC patients. The dotted line indicates the perfect prediction of the risk model. The solid line of pink indicates the performance of nomogram, and the dotted line closer to diagonal indicates better prediction.

### Analysis of the Association Between Risk Signature and Immunocyte Infiltration, Tumor Microenvironment

We quantified the quantity of immunocytes in every specimen using the “Cell-type Identification by Estimating Relative Subsets of RNA Transcripts (CIBERSORT)” algorithm (Newman et al., 2015). The single sample Gene Set Enrichment Analysis (ssGSEA) algorithm was employed to calculate 16 immunocyte infiltration and 13 immunity-related function scores for each KIRC sample. The diversities in immunocyte infiltration and immunofunction amongst diverse risk score subgroups were contrasted. We calculated the diversities in the expressing levels of immune checkpoint-related genes between diverse groups. The Tumor Immune Dysfunction and Exclusion (TIDE) algorithm was employed to assess the efficacy of potential immune checkpoint blocking (ICB) response (http://tide.dfci.harvard.edu) (Jiang et al., 2018). The tumor microenvironment (TME) scoring for every KIRC sufferer was quantified via the “Estimation of Stromal and Immune cells in Malignant Tumor tissues using Expression data (ESTIMATE)" algorithm (Yoshihara et al., 2013), including stroma scoring, immunoscore, and estimated scoring, and the potential differences in TME between different risk groups were estimated by comparing the diversities in TME scores between the risk_high_ group and risk_low_ group.

### Tumor Mutation Burden and Prediction of Potential Therapeutic Drug Sensitivity

We downloaded the somatic cell mutation data of KIRC tissue samples from the TCGA database (TCGA.KIRC.varscan.somatic.maf.gz). Tumor mutation burden (TMB) scoring was calculated based on somatic mutation data. The IC_50_ values of therapeutic medicines were calculated by the R package “pRRophetic”. The values of IC_50_ represent 50% inhibited cells, that is, the cell survival rate is half of the control sample, which is the corresponding drug concentration. The lower the IC_50_ value, KIRC patients are more sensitive to this drug.

### Analysis of Genes Associated With Immunity and Risk Score

R package “edgeR” was employed to screen for differentially expressed genes (DEGs) in the risk_high_ group and risk_low_ group (screening criteria: |log2FC| > 1, FDR < 0.05). As per the immunoscore in the TME scoring of each KIRC patient, KIRC patients were separated into 2 groups: the high-immunity group and the low-immunity group. The differentially expressed immune-related genes were obtained by the same method. Risk-immune-related genes were acquired via the intersection of risk differential genes and immune differential genes. The prognostic significance of these risk-immunity-associated genes was assessed by univariable Cox analyses. Spearman correlative analysis was employed to evaluate the association of prognostic risk-immunity-associated genes with immunocytes and immunofunction. Gene Ontology (GO), Kyto Encyclopedia of Genes and Genomes (KEGG) and Disease Ontology (DO) analyses were completed on risk-immunity-associated genes to explore the biological processes and potential signal pathways related to these genes (Chen et al., 2017). These prognostic risk-immune-related genes were applied to the construction of protein-protein interaction (PPI) networks through search tool for recurring instances of neighbouring genes (STRING) online database (medium confidence > 0.4), and then the hub genes were extracted from PPI networks using the CytoHubba plugin of Cytoscape (https://cn.string-db.org/cgi) (Zhou et al., 2021).

### Statistical Analysis

Log-rank test was used for the K-M approach to create the survival curve. The Chi-square test was utilized to determine the diversities between different data sets or different classified data, and the Wilcoxon-rank test was utilized to determine the diversities between these 2 groups. Spearman method was used for correlation analysis, and cox proportional regressive analysis was employed to evaluate the influence of gene expression and clinical features on the prognoses of sufferers. When *p* < 0.05, the above statistical methods had statistical significance. All analysis was conducted using R version 4.1.0 and the corresponding feature packages.

## Results

### The Expression Level of m7G-Related Genes and m7G-Related MicroRNAs

**FIGURE 2:**
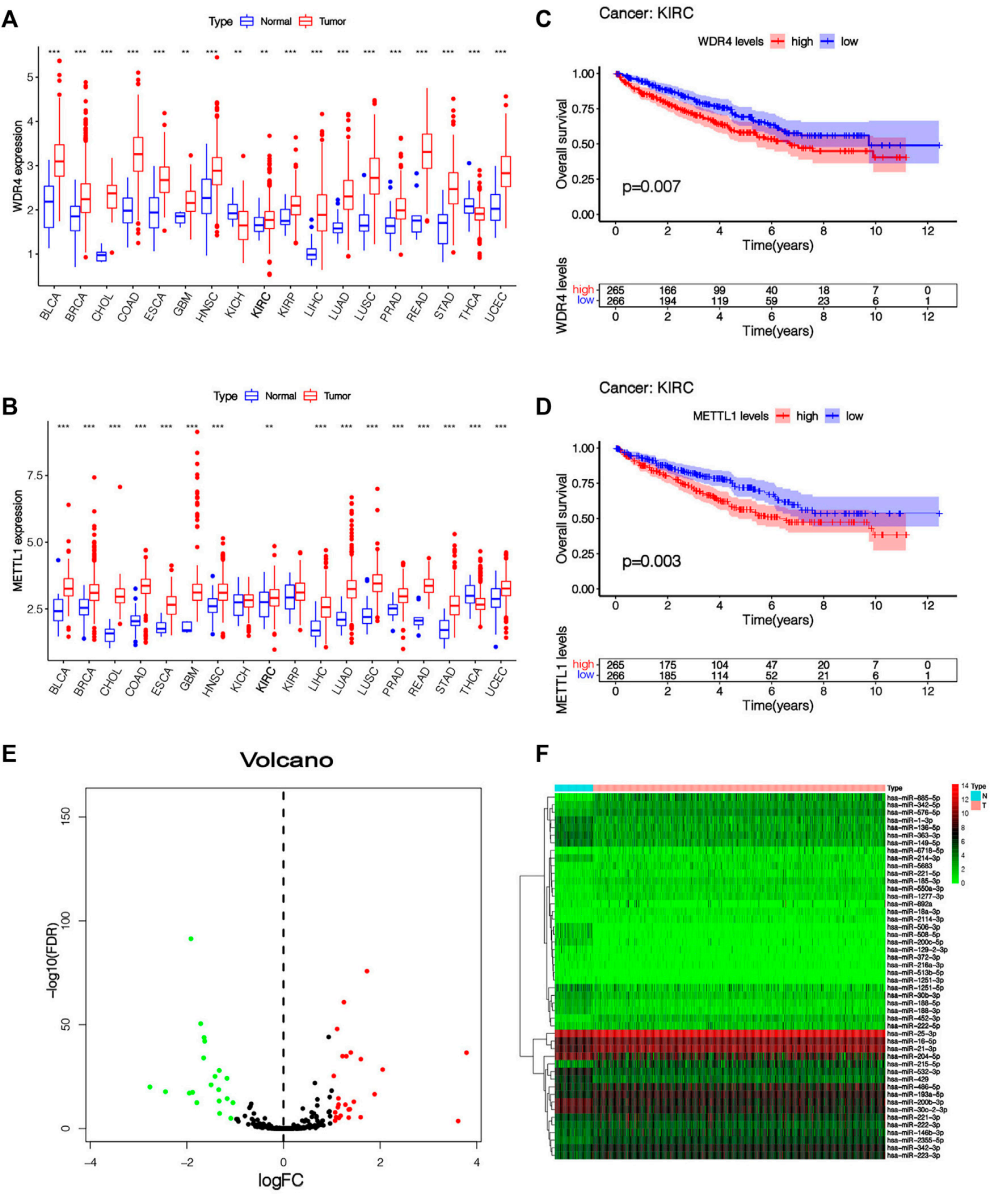
The expression level of m7G-related genes and m7G-related miRNAs. **(A,B)** Pan-cancer analysis of METTL1 and WDR4 in 18 tumor types. **(C,D)** K-M survival curve analysis of METTL1 and WDR4 in KIRC patients. **(E)** The volcanic plot of differentially expressed m7G-related miRNAs between KIRC tissue and normal tissue, which indicates that the distribution of log2FC and *p* values is reasonable. **(F)** Heatmap of 48 differentially expressed miRNAs expressed in KIRC tissue and normal tissue.

Our team studied the expressing level of 2 m7G-associated genes *METTL1* and *WDR4* in 18 tumor types and found that these two m7G-related genes were differentially expressed in many tumors, showing significant heterogeneity (Figures 2A,B). In KIRC, the expressions of *METT1* and *WDR4* in cancer samples were significantly greater vs. healthy samples (*p* < 0.01), the greater the expressing levels of *METT1* and *WDR4*, the worse the prognoses of KIRC sufferers (Figures 2C,D). The upstream miRNAs of these two m7G-related genes were predicted by the TargetScan online database (https://www.targetscan.org), and altogether 792 m7G-related miRNAs were acquired (Supplementary Table S1). The expression of these 792 m7G-related miRNAs in 545 KIRC samples and 71 normal samples was analyzed. Altogether 48 differentially expressed m7G-related miRNAs were acquired (|log2FC| > 1, FDR < 0.05), and the expressing levels of 22 miRNAs were low in cancer tissues while the expressing levels of 26 miRNAs were high in tumor tissues (Supplementary Table S2). The results are presented in the shape of a volcano plot (Figure 2E). The heatmap shows the expression of 48 differentially expressed m7G-related miRNAs (Figure 2F).

### Construction and Verification of the Prognostic Risk Model Based on m7G-Related MicroRNAs

**FIGURE 3:**
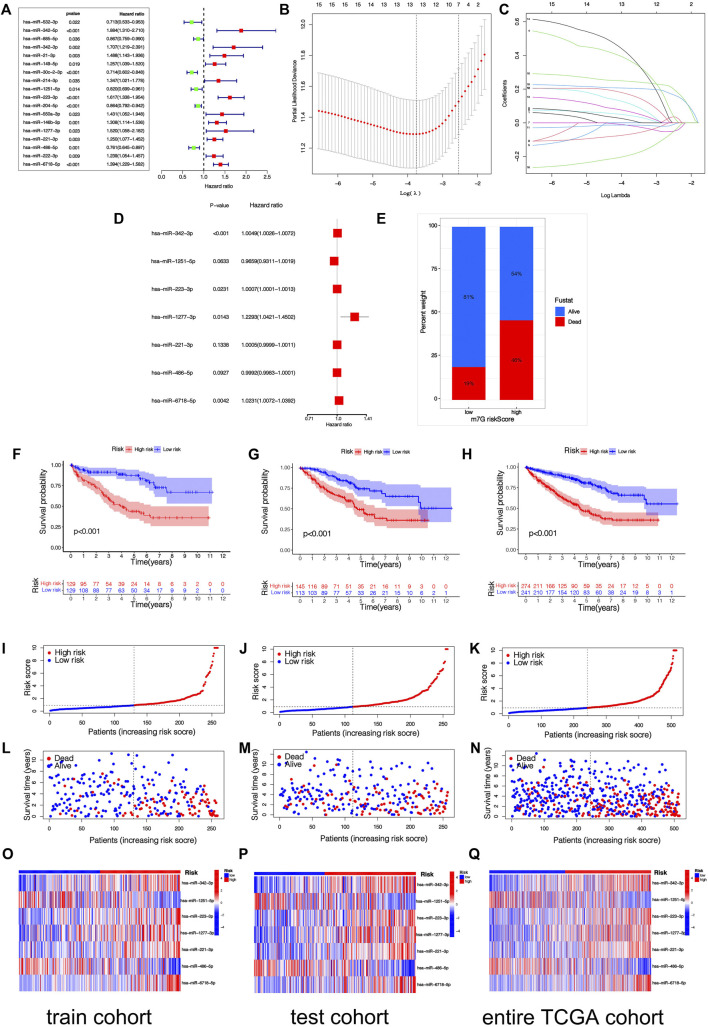
(Continued).

By taking the survival time as the prognostic index, a total of 516 patients with KIRC were used to construct and verify this model. Patients were stochastically separated into the training cohort and test cohort (Supplementary Table S3). In the training cohort, 48 differentially expressed m7G-related miRNAs were analyzed by univariate Cox regression, and altogether 18 m7G-related miRNAs with prognostic values were obtained (Figure 3A). Then, in order to optimize our model, LASSO regression analysis was performed on 18 prognostic m7G-related miRNAs to eliminate highly correlated prognostic miRNAs to avoid overfitting, and 13 most representative candidate miRNAs were found (Figures 3B,C). Cross-validation results show that LASSO regression analysis was the best. Multivariable Cox regressive analyses were completed on those 13 candidate miRNAs, and finally, 7 risk miRNAs and their corresponding coefficients were obtained, which were utilized to construct the risk signature (Figure 3D; Table2). The risk scoring of every KIRC sufferer can be computed as:

$$\begin{array}{l} \text{Risk score}\;=\;\left(\text{0}{\text{.}57247064179788\text{∗Exp}}_{\text{miR}-342-3\text{p}}\right)\\ \;\;\;\;\;\;\;\;\;\;\;\;\;\;\;\;\;\;\;\;\;+\;\left(-\text{0}{\text{.}130825723882467\text{∗Exp}}_{\text{miR}-1251-5\text{p}}\right)\\ \;\;\;\;\;\;\;\;\;\;\;\;\;\;\;\;\;\;\;\;\;+\;\left(\text{0}{\text{.}246195045745634\text{∗Exp}}_{\text{miR}-223-3\text{p}}\right)\\ \;\;\;\;\;\;\;\;\;\;\;\;\;\;\;\;\;\;\;\;\;+\;\left(\text{0}{\text{.}588243565862617\text{∗Exp}}_{\text{miR}-1277-3\text{p}}\right)\\ \;\;\;\;\;\;\;\;\;\;\;\;\;\;\;\;\;\;\;\;\;+\;\left(\text{0}{\text{.}276954654176782\text{∗Exp}}_{\text{miR}-221-3\text{p}}\right)\\ \;\;\;\;\;\;\;\;\;\;\;\;\;\;\;\;\;\;\;\;\;+\;\left(-\text{0}{\text{.}223588405707281\text{∗Exp}}_{\text{miR}-486-5\text{p}}\right)\\ \;\;\;\;\;\;\;\;\;\;\;\;\;\;\;\;\;\;\;\;\;+\;\left(\text{0}{\text{.}28936774470365\text{∗Exp}}_{\text{miR}-6718-5\text{p}}\right) \end{array}$$

**TABLE 2:** Seven candidate miRNAs and their corresponding coefficients were obtained by multivariate Cox regression analysis.

Candidate miRNAs	Coefficient
hsa-miR-342-3p	0.57247064179788
hsa-miR-1251-5p	−0.130825723882467
hsa-miR-223-3p	0.246195045745634
hsa-miR-1277-3p	0.588243565862617
hsa-miR-221-3p	0.276954654176782
hsa-miR-486-5p	−0.223588405707281
hsa-miR-6718-5p	0.28936774470365

**FIGURE 4:**
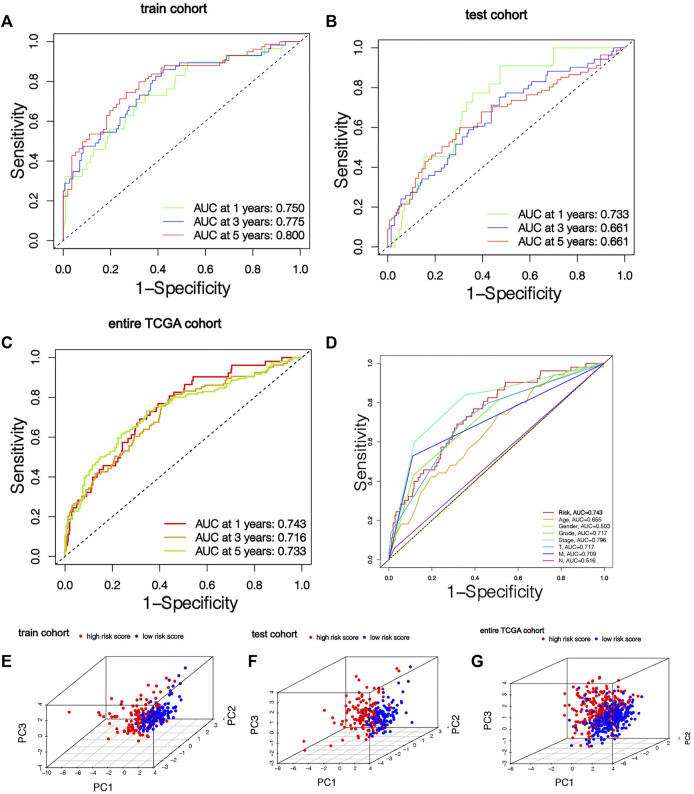
The risk signature has good prediction accuracy. **(A-C)** ROC curves of patients in training cohort, testing cohort and entire TCGA cohort based on risk score for 1, 3 and 5 years. **(D)** ROC curve based on risk score and clinical features in the entire TCGA cohort. **(E–G)** Training cohort, testing cohort and entire TCGA cohort PCA results show that risk score has the ability to divide KIRC patients into two subgroups.

Among the 7 risk miRNAs, 5 miRNAs (*miR-342-3p, miR-223-3p, miR-1277-3p, miR-221-3p, and miR-6718-5p*) with positive coefficients can be deemed as the factors of unsatisfactory prognoses of sufferers, i.e., the increased expression of these miRNAs is associated with the low OS rates of sufferers, whereas 2 miRNAs (*miR-1251-5p* and *miR-486-5p*) with negative coefficients may be used as KIRC inhibitors, i.e., the increased expression of these 2 miRNAs is beneficial to the prognoses of KIRC sufferers, which has been verified in the KM survival curve (Supplementary Figure S1). As per the mid-value of the risk scoring of the training cohort, all cohort patients were separated into the risk_high_ group and risk_low_ group. K-M survival analyses show that remarkable diversities existed in OS rates between different risk groups (Figures 3F–H). Overall, the OS rates of the risk_high_ group was lower vs. the risk_low_ group. Similarly, the mortality of KIRC sufferers in different groups were not similar, and the death rate of KIRC sufferers in the risk_high_ group was greater (Figure 3E). We ranked the risk scoring of sufferers, and the survival state and distribution of patient risk scores are shown in Figures 3I–N. With the increase in risk scores, the number of deaths of KIRC patients also increases. The heatmap shows the expression of 7 risk miRNAs. It can be seen that the 2 risk miRNAs, *miR-1251-5p* and *miR-486-5p*, are mostly under-expressed in risk_high_ sufferers (Figures 3O–Q). ROC curve was used to verify the accurateness of the risk signature in forecasting the prognoses of KIRC sufferers (Figures 4A–D). The AUC of 1, 3, and 5 years in the training cohort is 0.750, 0.775 and 0.800 separately, which shows that the model exhibits satisfactory accuracy in predicting patients’ survival. The results of principal component analysis (PCA) show that risk scoring has the ability to separate sufferers into the risk_high_ group and risk_low_ group (Figures 4E–G). The same verification results were acquired in the test cohort and full TCGA cohort.

### Association Between the Risk Signature and Clinical Features

**FIGURE 5:**
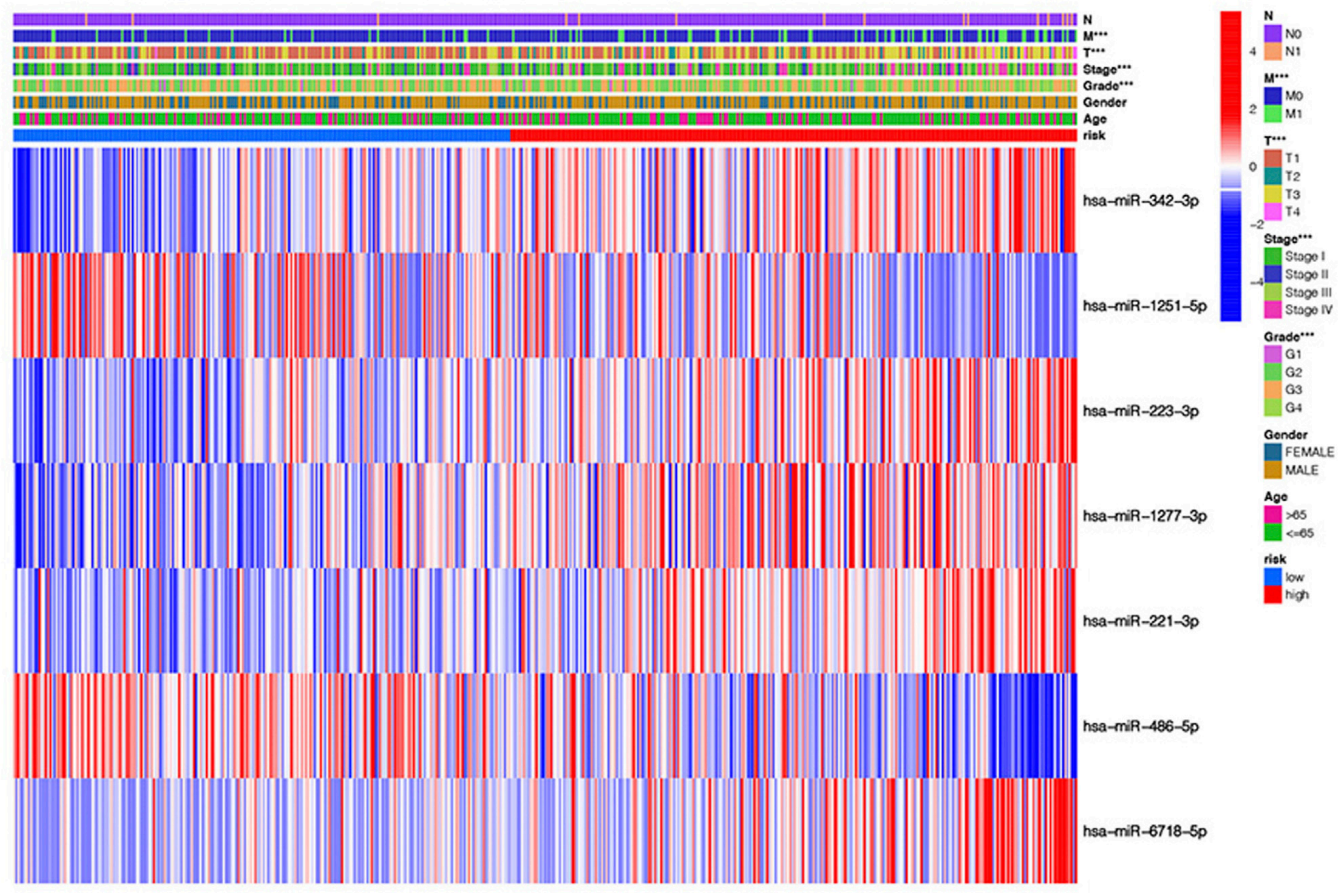
The heatmap shows the clinical characteristics and risk scoring of every KIRC sufferer.

**FIGURE 6:**
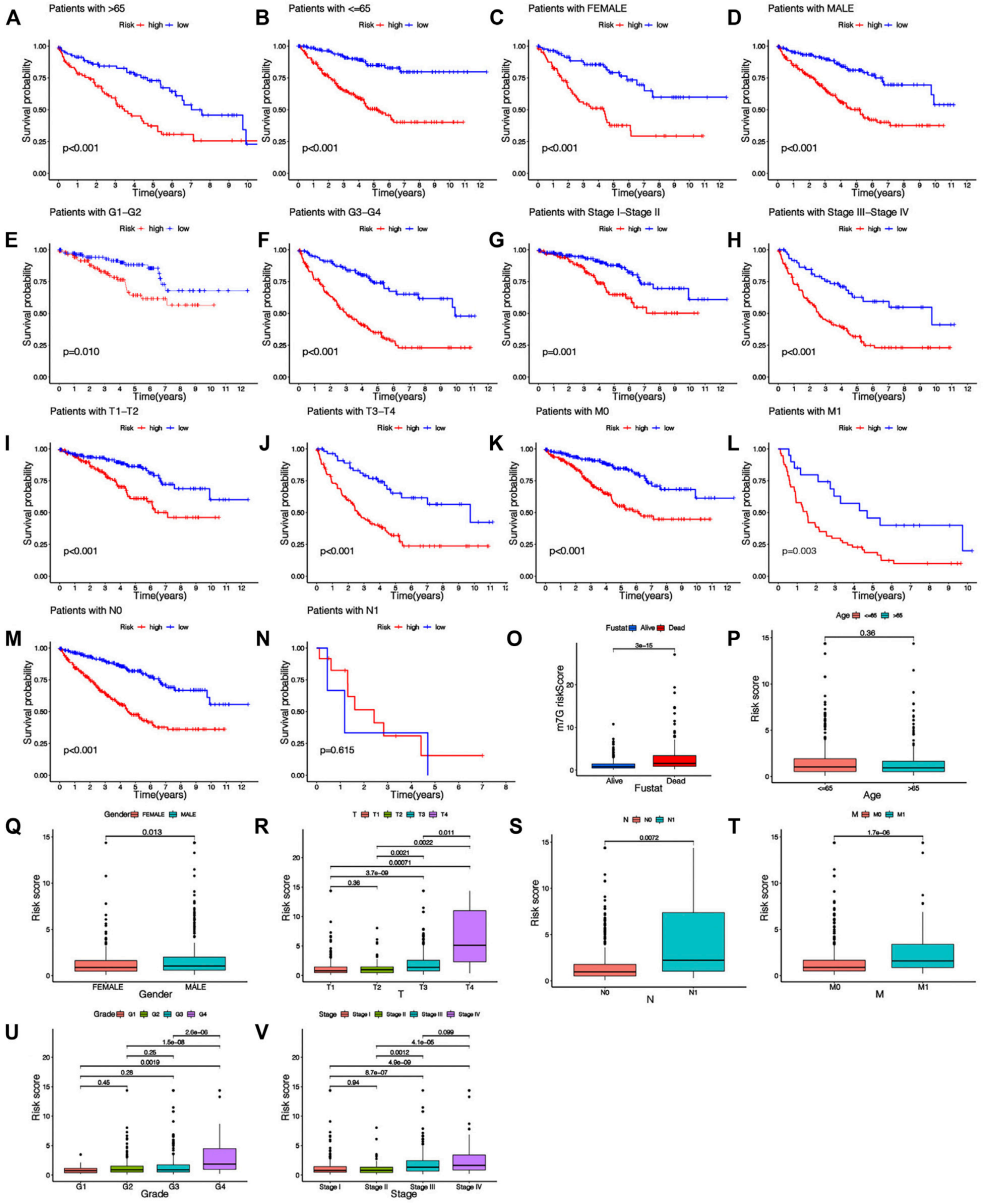
Relationship between risk score and clinical characteristics. **(A–N)** K-M survival curves for patients with different clinical features. **(O–V)** The histogram shows the distribution of risk scores among different clinical subgroups.

The heatmap shows the clinical characteristics and risk scoring of every KIRC sufferer (Figure 5). To further prove the clinical feasibility of the risk signature, we classified the sufferers according to different clinical characteristics. The K-M survival analysis results showed that except for sufferers with N1, survival diversities existed between the risk_high_ and risk_low_ groups in subgroups with different clinical features, and risk_low_ groups all had a better prognosis (Figures 6A–N). It showed that the prediction risk signature had wide clinical applicability. We also evaluated whether there were diversities in risk scores among KIRC sufferers with diverse clinical features. We can come to the conclusion that risk scoring was remarkably associated with a variety of clinical features (Grade, TNM staging, Stage) (Figures 6O–V). The risk scores of sufferers with G3-G4 were significantly higher than those with G1-G2, and those with T3-T4 were remarkably greater in contrast to those with T1-T2, and those with high pathological grades were remarkably greater in contrast to those with low pathological grades. The association between risk scoring and clinical features corresponds to prognoses, that is, sufferers with risk_high_ scores tend to have poor clinical characteristics and poor prognoses.

### Independence of the m7G-Related MicroRNAs Risk Score Regarding Other Clinical Factors

**FIGURE 7:**
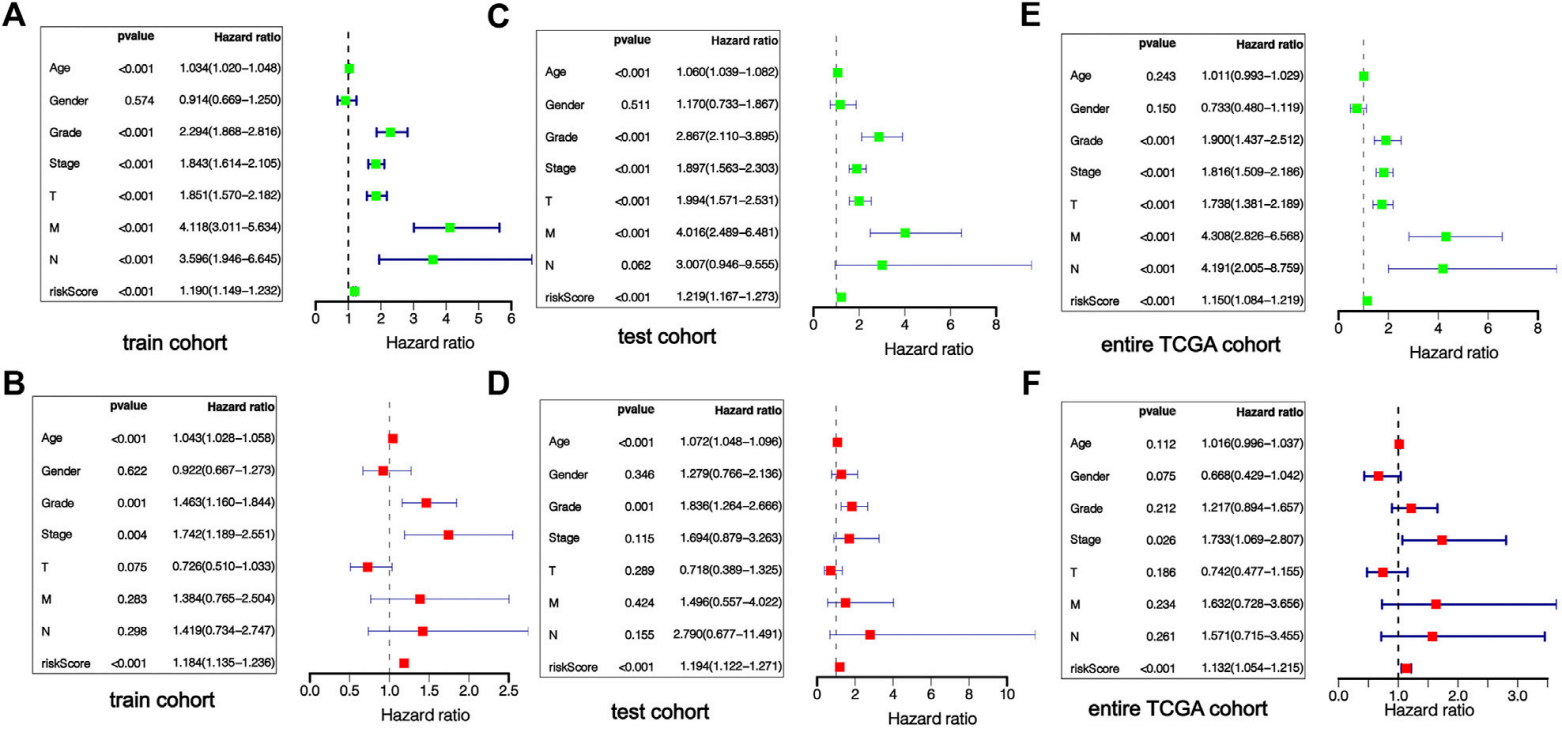
Independent verification of prognostic risk score. The forest map results of univariate and multivariate regression analysis show that risk score is an independent prognostic factor of KIRC patients, training cohort **(A,B)**, testing cohort **(C,D)**, entire TCGA cohort **(E,F)**.

Univariable-multivariable Cox regressive analyses were used to validate if the risk scoring was an independent predictor of the patient’s prognosis, regardless of clinical features. In the training cohort, univariable Cox results revealed that risk scoring was remarkably associated with the prognosis of sufferers (*p* < 0.01), and multivariable Cox regressive results (*p* < 0.01) further evidenced that risk scoring could serve as independent prognosis biomarkers in KIRC patients regardless of clinical characteristics (Figures 7A,B). The same univariate-multivariate Cox analysis results were obtained in the test cohort and full TCGA cohort (Figures 7C–F). The above results show that the prognosis-related risk scoring on the foundation of m7G-associated miRNAs has a certain significance for the prognosis evaluation of KIRC patients.

### Establishment and Verification of Nomogram

**FIGURE 8:**
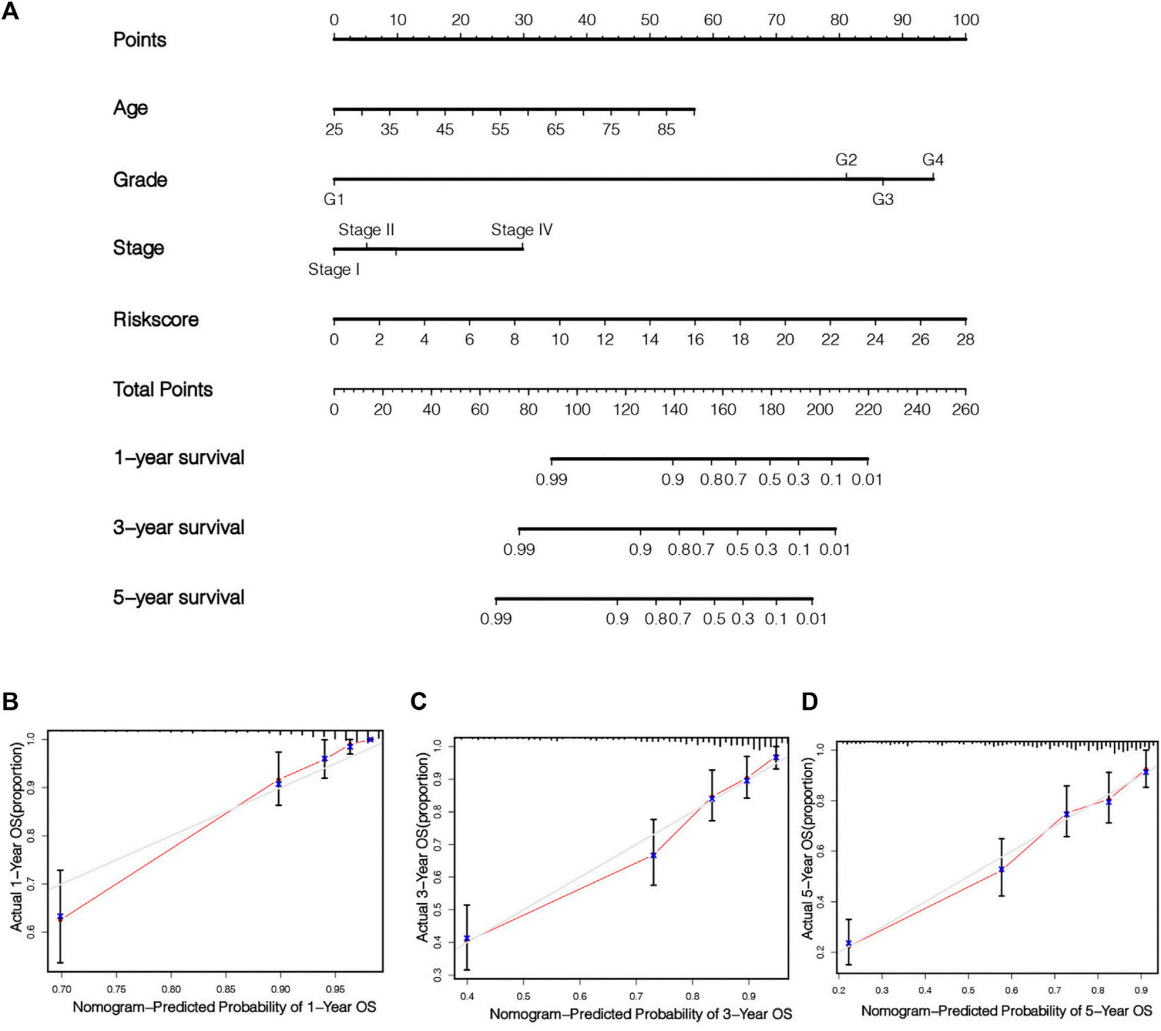
Establishment and verification of nomogram. Combined with age, stage, grade and risk score, a nomogram was established to quantitatively predict the 1, 3 and 5 years survival of KIRC patients **(A)**. The calibration chart illustrates the prediction ability of this nomogram **(B–D)**.

In order to fully exploit our prognostic risk signature clinically, a nomogram was established on the foundation of 3 clinical factors (age, grade, stage) and risk scoring for the quantitative prediction of 1-, 3-, and 5-year survival in KIRC patients (Figure 8A). Subsequently, the calibration curve was employed to verify the predictive ability and accurateness of the nomograph model (Figures 8B–D). The outcomes showed that nomogram had the ability to accurately estimate the OS of KIRC patients. It also shows that the nomogram has the value and potential to precisely forecast the prognoses of KIRC sufferers clinically.

### Tumor Microenvironment Analysis and Immune Infiltration Analysis

**FIGURE 9:**
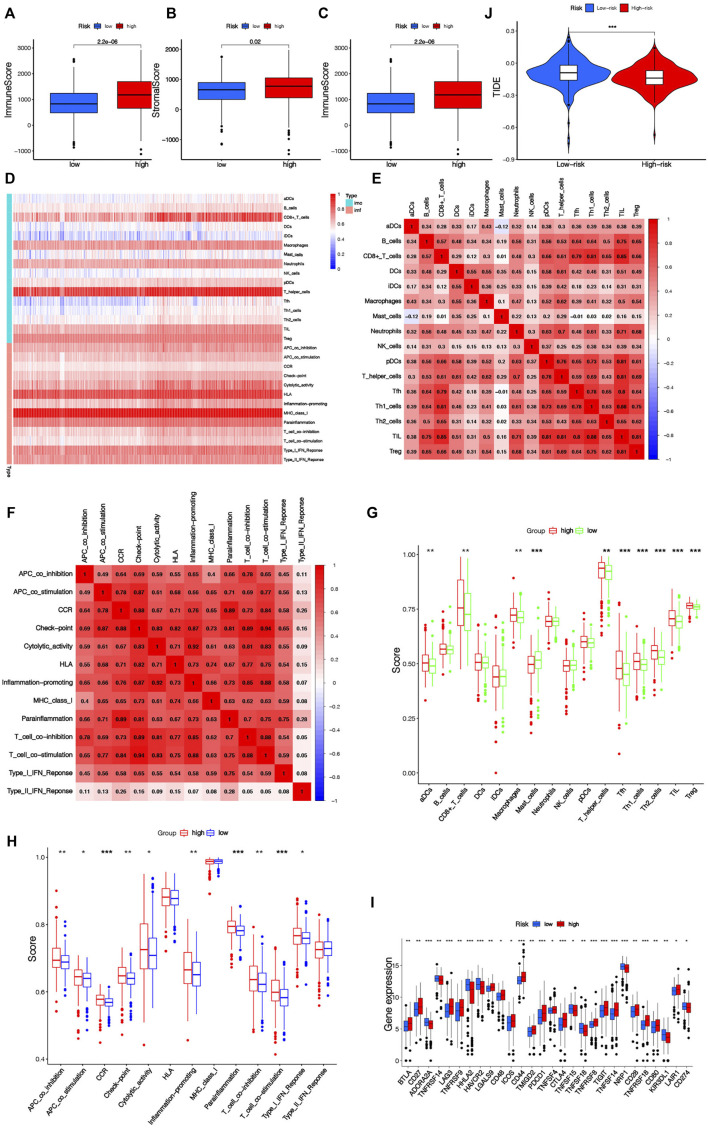
(Continued).

The TME score of each KIRC patient was obtained by “ESTIMATE” algorithm. It can be seen that remarkable diversities existed in TME scoring (including stroma scoring, immunoscore and estimated scoring) between the two risk groups (Figures 9A–C). The TME score of the risk_high_ group was higher, which suggested that there was a potential connection between risk scoring and the TME of KIRC sufferers. Figure 9D shows the infiltration of immunocytes and immunofunction in 516 KIRC samples, and the correlation between immunocytes and immunofunction is shown in Figures 9E,F. To evaluate the relationship between the risk scoring and immunity status of KIRC patients, ssGSEA was used to evaluate the diversity in immunocytes scoring and immune function scoring between the risk_high_ group and risk_low_ group. The scoring of CD8^+^ T cells, Mast cells, Macrophages and other immune cells was different between the risk_high_ group and risk_low_ group (Figure 9G). There were also remarkable diversities in the scores of immune-related pathways like Parainflammation, T cell costimulation, CCR, and APC co-suppression between the two risk groups (Figure 9H). It can be concluded that most immunocyte scoring and immunofunction scoring are greater in the risk_high_ group, and there are remarkable differences in immune infiltration levels among diverse groups. The expressing levels of common immune checkpoints in the risk_high_ group and risk_low_ group were also calculated, and it can be concluded that the expression of the majority of immune checkpoints, like CD27, CD48, CD44, CD28, CD274 (PD-L1), etc., is greater in the risk_high_ group (Figure 9I). The results of the above analyses suggest that the immunity status of risk_high_ sufferers may be more active. At the same time, the TIDE algorithm was leveraged to assess the underlying ICB treatment reaction. The TIDE scoring of risk_high_ sufferers was lower vs. risk_low_ sufferers, which suggested that risk_low_ sufferers displayed the potential of immune escape (Figure 9J). By comparison with risk_low_ sufferers, the effect of ICB treatment in risk_high_ sufferers may be more significant. It further shows that our risk signature has a great prospect in guiding individualized immunotherapy for KIRC patients.

### Tumor Mutation Burden and Prediction of Potential Drug Sensitivity of KIRC

**FIGURE 10:**
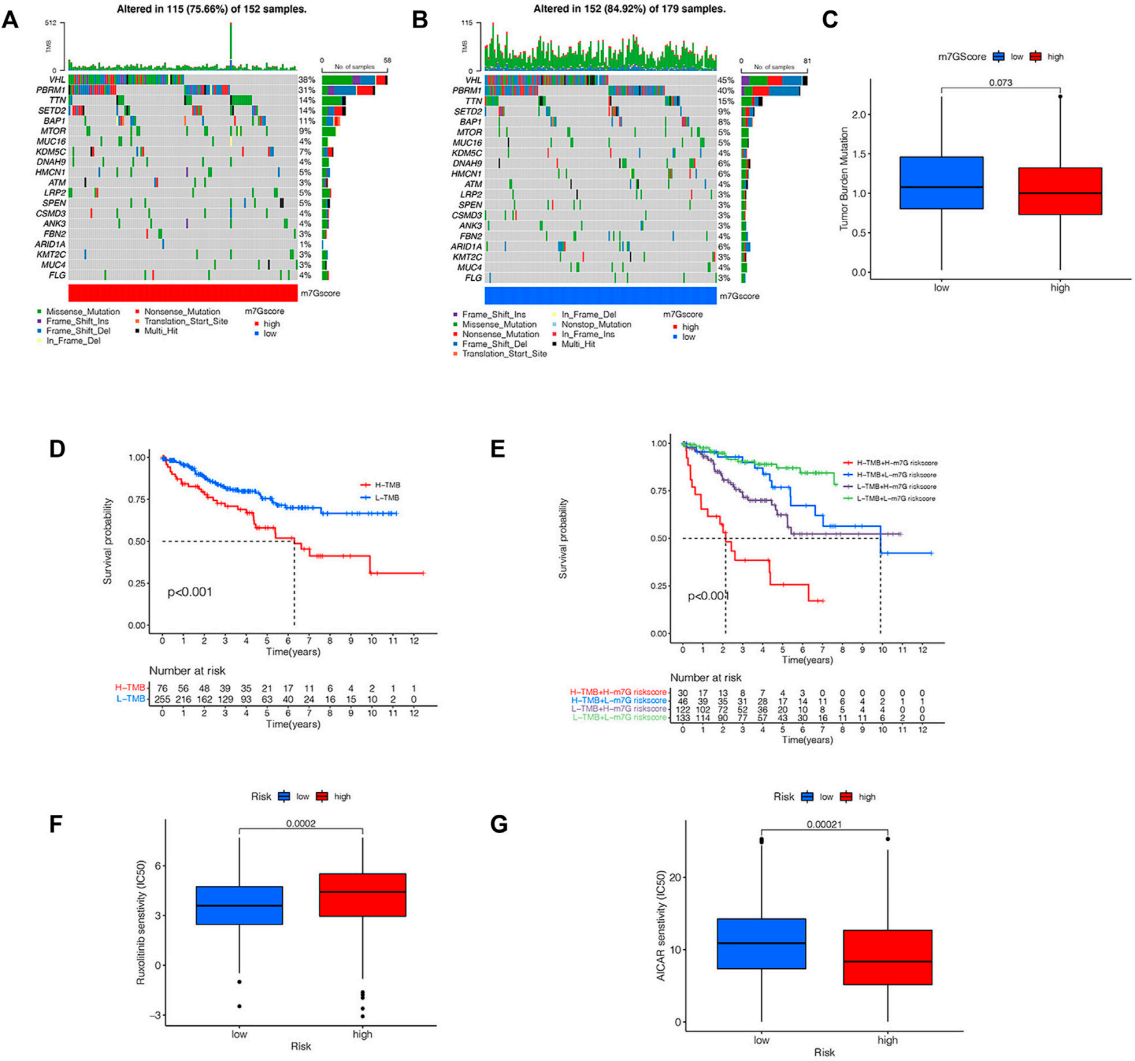
Analysis of TMB and drug sensitivity. **(A,B)** The waterfall plot shows the somatic mutation characteristics of two risk groups. **(C)** Difference of TMB between two risk groups. **(D)** K-M survival analysis of high-TMB group and low-TMB group. **(E)** K-M survival analysis based on TMB and risk score. **(F,G)** IC_50_ values of AICAR and Ruxolitinib in two risk groups.

We analyzed the variation of somatic cell mutation in two risk groups. An overall 84.9% risk_low_ sufferers displayed the mutation, whereas only 75.66% risk_high_ sufferers displayed such mutation (Figures 10A,B). Although the TMB is different among different risk groups, it is not significant (Figure 10C). In risk_high_ sufferers, the most frequently mutated gene is VHL and the most frequent mutation type is a missense mutation, while in risk_low_ sufferers, the most frequently mutated gene is VHL and the most frequent mutation type is Frame-Shift-Ins. As per the outcomes of the K-M analyses, the prognoses of high-TMB sufferers are poorer vs. low-TMB sufferers (Figure 10D). In addition, we combined TMB and risk scores in KIRC patients for survival analyses, and conclude that sufferers with high-TMB and risk_high_ scores have the worst prognosis (Figure 10E), which further verifies the ability of our risk signature in forecasting the OS of KIRC sufferers. By comparing the IC_50_ values of common drugs in different risk groups, the sensitivity of KIRC patients to potential therapeutic drugs can be predicted. There are significant differences in the IC_50_ values of 14 drugs (MS-275, AUY922, CH5424802, YM201636, CCT018159, CCT007093, NU-7441, AICAR, THZ-2-49, Genentech Cpd 10, BX-912, Ruxolitinib, GSK1904529A, FH535) in the risk_high_ group and risk_low_ group (Supplementary Figure S2), and the IC50 value of AMPK activator (AICAR) in risk_high_ sufferers is lower (Figure 10G), which suggests that AICAR has a more significant effect in risk_high_ sufferers. The IC_50_ value of JAK1/2 inhibitor (Ruxolitinib) in risk_low_ patients is lower (Figure 10F), which indicates that the therapeutic effect of Ruxolitinib is more beneficial in risk_low_ people. The prediction of the efficacy of these potential therapeutic drugs can be helpful for the clinical drug treatment of KIRC. These results also demonstrate that our risk signature displays certain significance in guiding the drug therapy for KIRC sufferers.

### Analysis of Seven Risk MicroRNAs in the Predicted Signature Model

**FIGURE 11:**
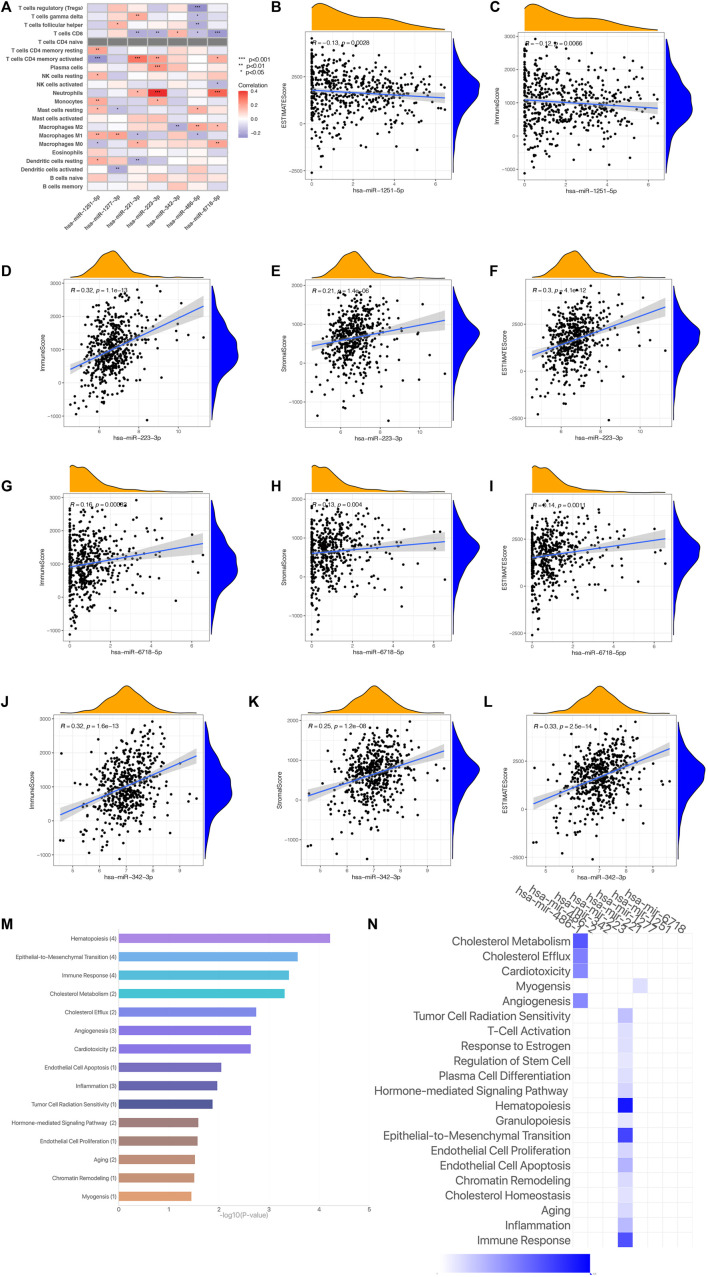
(Continued).

As we have come to the conclusion that the risk scoring may be remarkably associated with the immune status and TME of KIRC patients. We will perform further research on the relationship among the expression of Seven risk miRNAs and immune status and TME. Figure 11A shows the correlation between Seven risk miRNAs and 22 kinds of immune cells, among which neutrophils and T cells CD8 are closely related to risk miRNAs. The expression of Seven miRNAs and TME score are shown in Figures 11B–L. The outcomes reveal that the expressing level of *miR-1231-3p* is related to immunity scoring and estimated scoring in a negative manner (*p* < 0.05) (Figures 11B,C). With the increase of the expressing levels of *miR-1231-3p*, *miR-1227-3p* and *miR-342-3p*, the stromal scoring, immunoscore and estimated scoring of KIRC patients gradually increase (*p* < 0.05) (Figures 11D–L). The enrichment analysis of 7 risk miRNAs based on the miRNA function analysis website (http://www.lirmed.com) shows that these risk miRNAs are mainly enriched in relevant functional pathways such as hematopoiesis, epidemiological-to-immunological transition, immune response, angiogenesis, etc. (Figures 11M,N). The potential functional pathways by which risk scores affect the occurrence and development of KIRC are expounded from another angle. These results provide certain new supports for the accuracy and reliability of our prognostic risk signature.

### Screening Process for Hub Prognostic Genes and the Identification of Prognostic Genes Highly Related to m7G-Related MicroRNA Risk Score and Immunity

**FIGURE 12:**
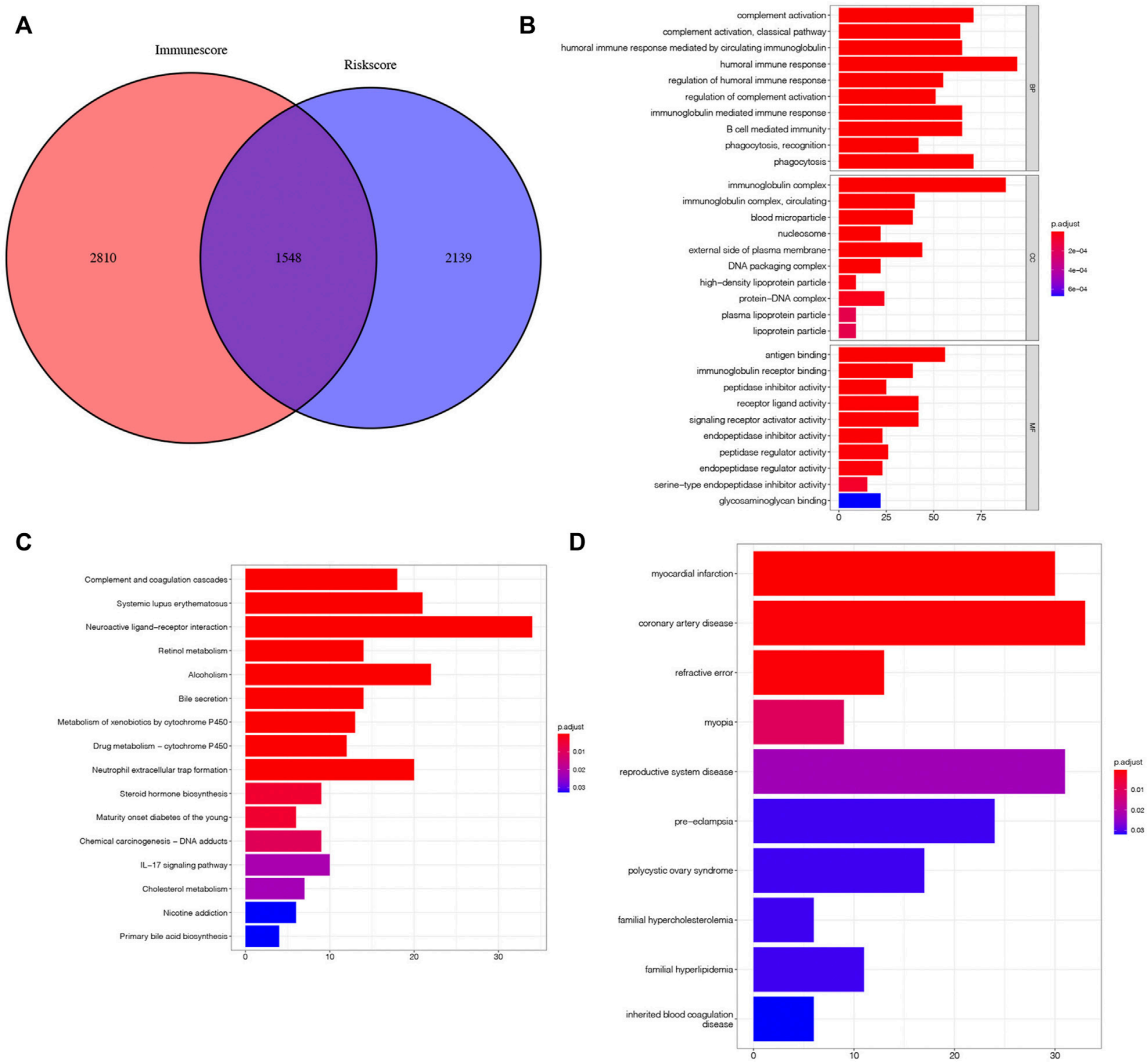
Search for risk-immune-related genes. **(A)** Intersection gene of risk score and immune score. **(B–D)** GO, KEGG, DO enrichment analysis of prognostic risk-immune-related genes.

**FIGURE 13:**
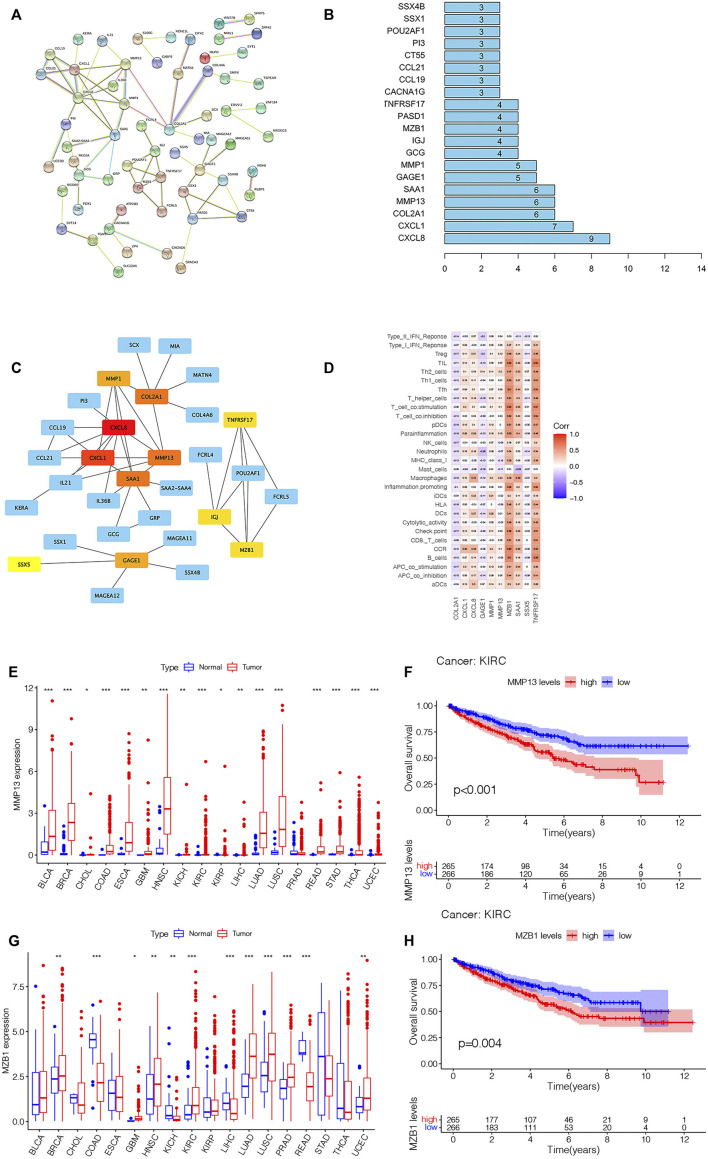
(Continued).

To further explore the genes affecting the prognoses of KIRC sufferers at the m7G and immune levels. Based on the risk score, an overall 3,687 DEGs were determined in the risk_high_ group and risk_low_ group in a complete cohort (screening criteria: |log2FC| > 1, FDR < 0.05). Based on the immune score of TME scoring, 4,358 immune-related DEGs were determined in the high-immunoscore group and low-immunoscore group (screening criteria: |log2FC| > 1, FDR < 0.05). By crossing the two DEGs, a total of 1,548 DEGs related to risk score and immune score were obtained (Figure 12A; Supplementary Table S4). Afterwards, the prognosis significance of those DEGs was studied *via* univariable Cox regressive analysis, and altogether 202 prognostic risk-immune-related genes were obtained (Supplementary Table S5). In order to study the biofunctions and pathways associated with risk scoring and immunity, these 202 prognostic risk-immune-related genes were analyzed by GO enrichment, KEGG pathway and DO analyses (Figures 13B–D). The results revealed that the majority of these genes were enriched in immunity-associated pathways and biofunctions. To further select the hub prognostic genes, through the STRING database, 71 genes were selected from 202 prognostic risk-immune-related genes to construct the PPI network (medium confidence >0.4), which contained 71 nodes and 72 edges (Figure 13A). Figure 13B shows the degree of correlation between genes, and *CXCL8* exhibits the strongest association with other genes. Then, the gene relationships were imported into Cytoscape. According to the cytoHubba plugin in Cytoscape, the top 10 hub genes were screened out, and we studied the hub genes (Figure 13C). Figure 13D shows the association among these hub prognosis risk-immunity-associated genes and immunocytes and immunofunction. The expression of *MMP13*, *MZB1* in 18 tumor types showed that *MMP13*, *MZB1* was differentially expressed in various tumors, and it can be seen that the expressing level of *MMP13*, *MZB1* in KIRC samples was greater vs. the corresponding normal samples (Figures 13E,G). K-M survival curve showed that the expressing level of *MMP13*, *MZB1* was remarkably associated with the OS of KIRC sufferers (Figures 13F,H).

## Discussion

KIRC is a heterogeneous disease. Even KIRC patients with similar tumor stages and clinical stages could display fairly different tumor progression and prognosis. Although there are many treatment methods (surgery, targeted drug therapy, immunotherapy) that can improve the OS rates of patients with KIRC to a certain extent, the prognosis of patients with advanced KIRC is still not satisfactory. Some of the existing biomarkers of KIRC that have been verified have very limited predictive ability, which is not enough to meet the needs of clinical diagnosis, treatment and the prediction of patient survival. Hence, it is imperative to find a biomarker which can precisely forecast the prognoses of KIRC sufferers and provide guidance and assistance for individualized treatment to avoid under-treatment and over-treatment.

M7G is a modification method of miRNA, which is related to the initiation of miRNA biogenesis, cell migration, tumor immunity and other biological processes. Recent studies and literature reports have shown that the abnormal expression of miRNA can affect the progress of many kinds of tumors, and that it is tightly associated with the prognosis of patients. Currently, there are many signatures based on miRNA that can precisely forecast the OS rates of sufferers, for example, m6A-related miRNAs signature can be utilized as a marker to forecast the prognoses of esophageal carcinoma patients (Li et al., 2021); the signature based on miRNA can accurately predict the survival of HNSCC sufferers (Zhao and Cui, 2020); the risk signature composed of necroptosis-related miRNAs can be utilized as a tool to accurately forecast the prognoses of sufferers with KIRC and colon cancer and guide immunotherapy (Huang et al., 2021; Chen W. et al., 2022); and the signature composed of nine immune-related miRNAs can predict the OS rates of patients with gastric cancer (Xu et al., 2021). *METTL1* and *WDR4* that are related to m7G modification on miRNA have been proved to be tightly associated with the onset of various diseases, and they are differentially expressed in various tumors including KIRC. In addition, they are also remarkably associated with the OS rates of KIRC sufferers. However, whether the m7G-related miRNAs signature can be utilized as a prognosis index for KIRC sufferers has not been elucidated.

Based on m7G-related miRNAs, our team established a prognostic risk signature comprising 7 m7G-associated miRNAs by univariable Cox, LASSO and multivariable Cox analyses. And a risk scoring computation equation was obtained to compute the risk scoring of every KIRC sufferers. In order to further assess the ability of the siganture in forecasting the prognoses of KIRC sufferers, it can be concluded by survival analyses that the OS of risk_high_ sufferers is lower vs. risk_low_ sufferers. Then, the ROC curve proved the accurateness of the signature in forecasting the prognoses of sufferers. Univariable-multivariable Cox analyses revealed that the risk score could be utilized as an independent prognostic index for KIRC patients. All these results were verified in the test cohort and full TCGA cohort. In addition, to facilitate the clinical application of signature risk score, our team constructed a nomogram to forecast the survival of KIRC sufferers in a quantitative manner. These results show that our prediction risk signature has great clinical application value in evaluating the prognosis of KIRC sufferers.

Our team also discussed the association among risk scoring and tumor microenvironment, common immune checkpoints. Because of the diversities in TME in diverse groups, these differences can promote the proliferative, migratory and invasive abilities of KIRC, which also explains the significant differences in prognoses amongst diverse groups. It can be seen that most risk_high_ patients have a high level of immunocyte infiltration, and the expressing levels of common immune checkpoint-associated genes in most risk_high_ patients are higher vs. risk_low_ patients. It indicates that the immune activity of risk_high_ sufferers is higher. In addition, by comparison with risk_high_ sufferers, the TIDE scoring of risk_low_ sufferers is lower, which suggests that risk_low_ sufferers have higher immune escape potential. This indicates that risk_high_ sufferers can benefit from ICB therapy. These results reveal that risk_high_ sufferers may benefit more from immune therapy. These immune analysis results can be helpful for guiding individualized immunotherapy for KIRC patients. In addition, we also analyzed the difference in TMB between the risk_high_ and risk_low_ groups, and the TMB of risk_low_ sufferers was higher vs. risk_high_ sufferers. Although this result was not statistically significant, it also explained the relationship between signature based on m7G-related miRNAs and TMB to some extent. We also tried to facilitate the clinical instruction of KIRC drug treatment based on our signature. By comparing the IC_50_ values of common drugs in different risk groups to speculate the potential drug sensitivity, it can be seen that AMPK activator (AICAR) is more suitable for risk_high_ sufferers, while JAK1/2 inhibitor (Ruxolitinib) has lower IC50 values in risk_low_ sufferers, which indicates that the therapeutic effect of Ruxolitinib is more beneficial to risk_low_ sufferers. The prediction of the efficacy of these potential drugs can be helpful for the individualized drug treatment of KIRC.

We further analyzed the influence of the expression of seven risk miRNAs that make up the prognosis signature on the patients with KIRC, and the results show that among these seven risk miRNAs, the expressing levels 5 miRNAs (*miR-342-3p, miR-223-3p, miR-1277-3p, miR-221-3p,* and *miR-6718-5p*) in cancer samples are greater in contrast to healthy samples. These 5 risk miRNAs can be considered risk factors for KIRC patients. The expressing levels of *miR-1251-5p* and *miR-486-5p* are low in cancer samples, which can be regarded as protective factors for patients with KIRC. And the results of K-M survival curve verify our conclusion. There are also some literature reports about the roles of these 7 risk miRNAs in tumorigenesis and development. For example, over-expressed *miR-1251-5p* can inhibit the proliferative, migratory and immunoescape-related abilities of KIRC cells (Yue et al., 2022), and over-expressed *miR-486-5p* can repress the proliferative ability and promote the apoptosis of KIRC cells (He et al., 2019), while *miR-223-3p* can facilitate the proliferative and metastatic abilities of renal clear cell carcinoma by downregulating SLC4A4 (Xiao et al., 2019). These functionally validated results further provide supports for our study: *miR-1251-5p* and *miR-486-5p* can act as protective factors for KIRC; highly expressed *miR-1251-5p* and miR-486-5p can prolong the survival of KIRC patients; *miR-223-3p* is a risk factor for KIRC; and the high expression of *miR-223-3p* can cause poor prognoses in KIRC patients. Although the other 4 risk miRNAs are not reported in KIRC, they are reported in other tumor diseases. For example, *miR-342-3p* can stimulate the malignant potential of NSCLC by regulating the expression of *LASP1* (Shen et al., 2020), and *miR-221-3p* can promote angiogenesis in cervical cancer by regulating the expression of THBS2 (Wu et al., 2019). Most of these seven risk miRNAs have certain influences on the progression of KIRC and other tumors, so the signature based on these seven risk miRNAs is convincing in forecasting the prognoses of sufferers. Meanwhile, we need further *in vivo* and *in vitro* assays to explore the causal link and function of risk miRNA, which are rarely reported in KIRC. With the increasing popularity of targeted therapy in recent years, nowadays, the targeted therapy of KIRC is also developing persistently, which brings us a hint that these seven risk miRNAs may foster the targeted therapy of KIRC.

Finally, based on the predicted signature risk score and the immunoscore, we obtained the risk-immunity-associated genes and analyzed the influence of these genes on the prognoses of KIRC sufferers. Subsequently, our team established a PPI net and screened out 10 hub genes. These hub genes may affect the progress of KIRC through m7G modification on miRNA and immune-related ways. In the future, we can focus on the mechanism and function of these hub genes affecting the progress of KIRC at the m7G level and immune level, providing new assistance to the early diagnosis and treatment of KIRC.

The aforesaid results show that the risk signature of prognosis prediction based on m7G-related miRNAs has reliable predictability and sensitivity. Comparing our prediction model with the published KIRC prognosis prediction model, we come to the conclusion that our model is superior to other KIRC prognosis prediction models. Generally speaking, the signature based on m7G-related miRNAs can precisely forecast the prognoses of sufferers and can foster the diagnosis and personalized treatment of KIRC. However, our research still has great limitations. First of all, our signature is only verified internally, and no suitable external data set has been found in the published database for the purpose of further evaluating the reliability of our signature. Secondly, we need further functional experiments to verify the relationship between risk miRNAs and m7G modification. Finally, as our research content is retrospective, we need to further verify our conclusion via *in vivo* and *in vitro* assays in the next stage. Although there are some shortcomings, it is the first time that a signature based on m7G-miRNAs can precisely forecast the prognoses of KIRC sufferers, hence, the present research is quite promising in terms of clinical practice.

## Conclusion

Our team smoothly established a prognosis-related risk signature on the foundation of m7G-associated miRNAs and verified its accuracy and reliability. A nomogram that could forecast the OS of KIRC sufferers in a quantitative manner was also established. The influence of risk score on the immune microenvironment and immunocyte infiltration of KIRC sufferers was also discussed. Our research results can accurately evaluate the prognosis of KIRC patients and facilitate individualized immunotherapy for KIRC patients. In addition, the identified 7 risk miRNAs are expected to be therapeutic targets for KIRC patients.

## Data Availability

The original contributions presented in the study are included in the article/Supplementary Material, further inquiries can be directed to the corresponding authors.

## Abbreviations

AUC, the area under the ROC curve; CIBERSORT, Cell-type Identification by Estimating Relative Subsets of RNA Transcripts; DEGs, differentially expressed genes; DO, Disease Ontology; ESTIMATE, Estimation of STromal and Immune cells in MAlignant Tumor tissues using Expression data; FDR, False Discovery Rate; GO, Gene Ontology; ICB, immune checkpoint blocking; KEGG, Kyto Encyclopedia of Genes and Genomes; KIRC, kidney renal clear cell carcinoma; K-M, Kaplan-Meie; Log FC, Log Fold Change; m7G, N7-methylguanosine; miRNAs, MicroRNAs; OS, overall survival; ROC, receiver operating characteristic; ssGSEA, single sample Gene Set Enrichment Analysis; TCGA, The Cancer Genome Atlas; TIDE, Tumor Immune Dysfunction and Exclusion; TMB, Tumor mutation burden; TME, tumor microenvironment.
