# Supplementary figures and images for: A Novel M7G-Related MicroRNAs Risk Signature Predicts the Prognosis and Tumor Microenvironment of Kidney Renal Clear Cell Carcinoma
Source: Front Genet. 2022 Jun 24;13:922358. doi: 10.3389/fgene.2022.922358 (PMC9263547; doi:10.3389/fgene.2022.922358)

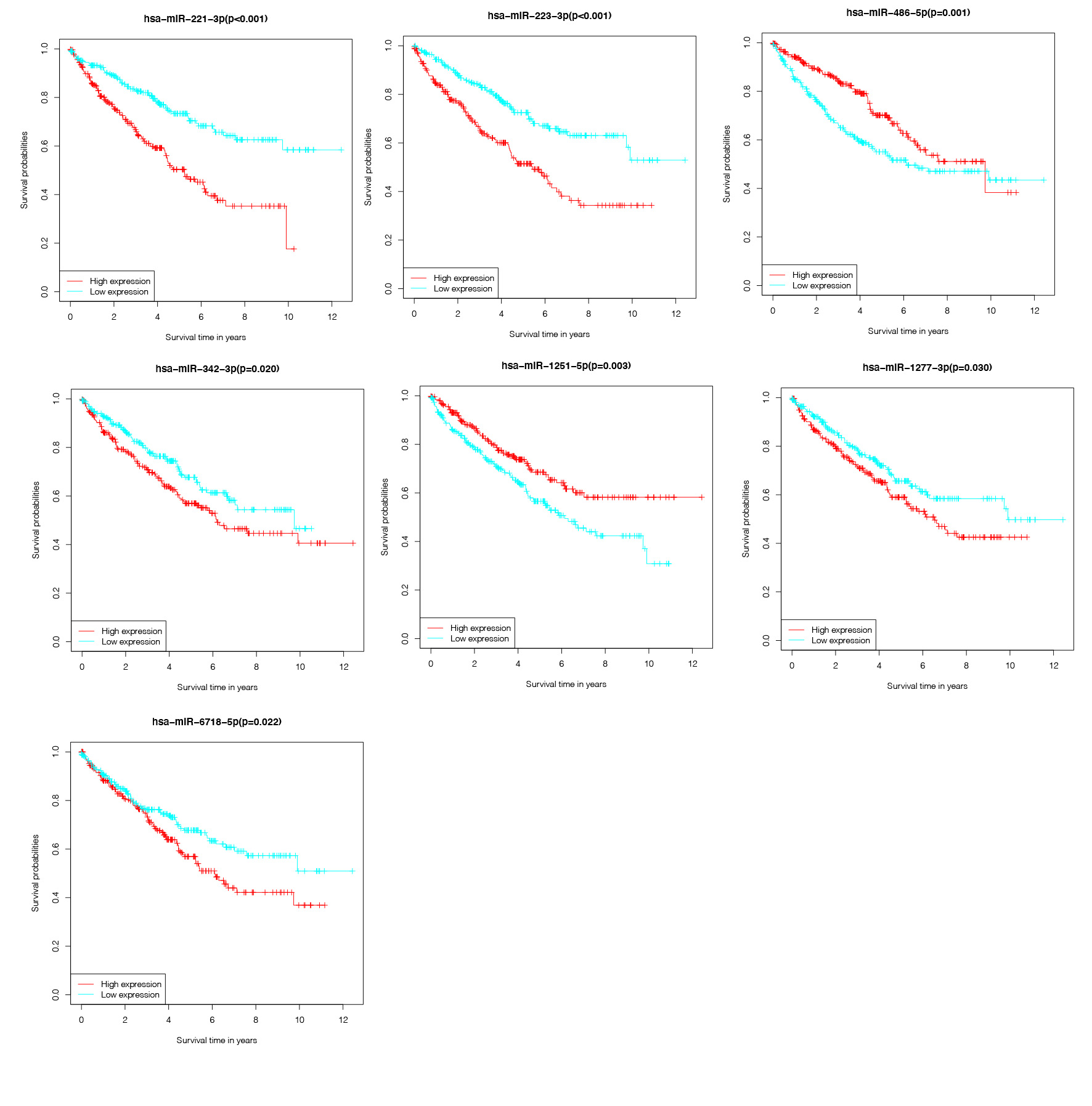

Supplement: Supplementary file 4 [file Image1.JPEG]

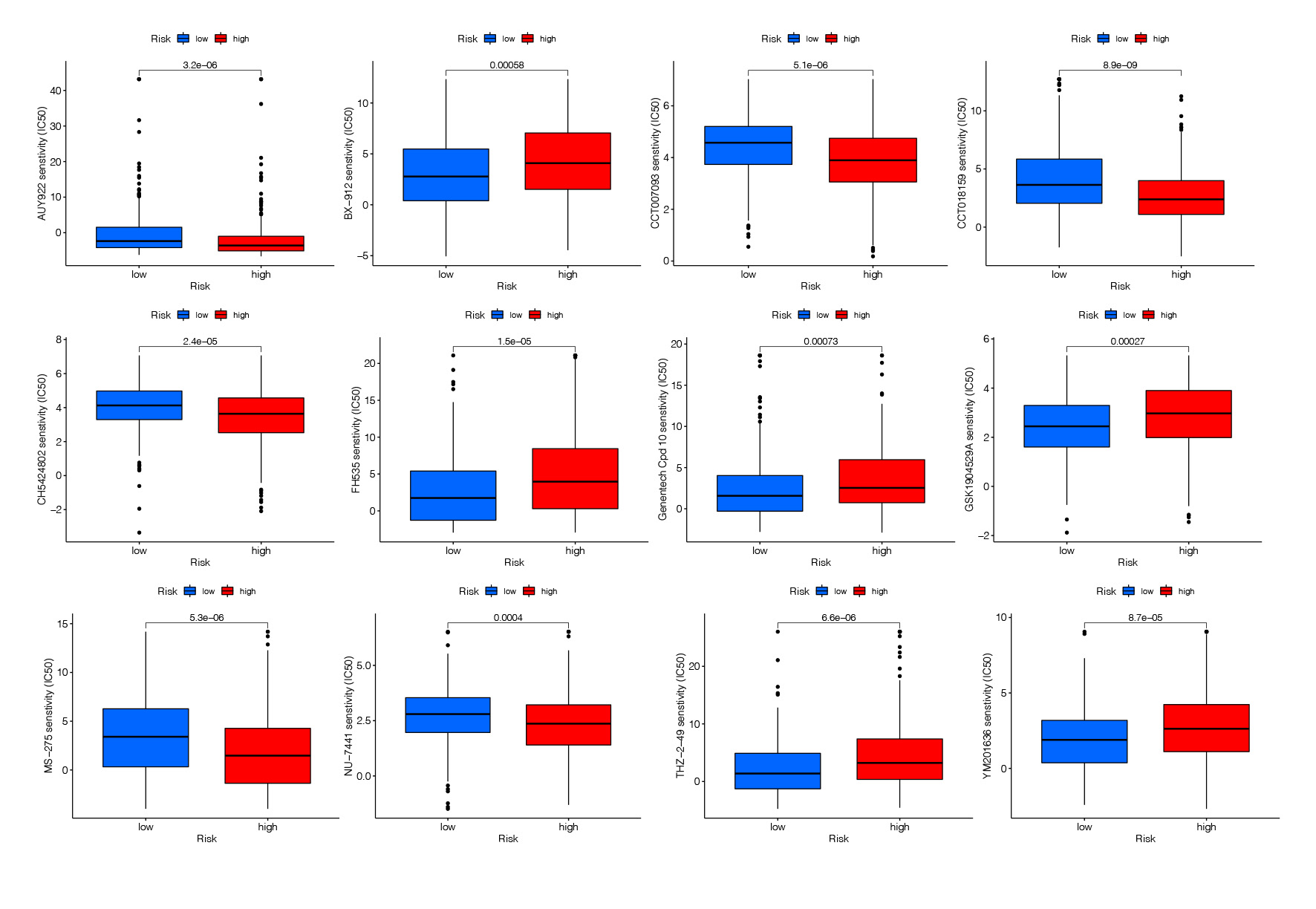

Supplement: Supplementary file 6 [file Image2.JPEG]
