# Supplementary material for: A Novel M7G-Related MicroRNAs Risk Signature Predicts the Prognosis and Tumor Microenvironment of Kidney Renal Clear Cell Carcinoma
Source: Front Genet. 2022 Jun 24;13:922358. doi: 10.3389/fgene.2022.922358 (PMC9263547; doi:10.3389/fgene.2022.922358)
Supplement: Supplementary file 8 [file Table6.DOCX]

**Supplementary Figure 1：**K-M analysis of the influence of 7 risk miRNAs expressions on the survival of KIRC patients.

**Supplementary Figure 2：**IC50 values of 12 common therapeutic drugs in different risk groups.
